# Functional Potential of Soil Microbial Communities in the Maize Rhizosphere

**DOI:** 10.1371/journal.pone.0112609

**Published:** 2014-11-10

**Authors:** Xiangzhen Li, Junpeng Rui, Jingbo Xiong, Jiabao Li, Zhili He, Jizhong Zhou, Anthony C. Yannarell, Roderick I. Mackie

**Affiliations:** 1 Energy Biosciences Institute, University of Illinois at Urbana-Champaign, Urbana, IL, United States of America; 2 Key Laboratory of Environmental and Applied Microbiology, CAS, Environmental Microbiology Key Laboratory of Sichuan Province, Chengdu Institute of Biology, Chinese Academy of Sciences, Chengdu, Sichuan, China; 3 Key Laboratory of Mountain Ecological Restoration and Bioresource Utilization, CAS, Ecological Restoration Biodiversity Conservation Key Laboratory of Sichuan Province, Chengdu Institute of Biology, Chinese Academy of Sciences, Sichuan, PR China; 4 School of Marine Sciences, Ningbo, Zhejiang, China; 5 Institute for Environmental Genomics and Department of Microbiology and Plant Biology, University of Oklahoma, Norman, OK, United States of America; 6 Department of Natural Resources and Environmental Sciences, University of Illinois at Urbana-Champaign, Urbana, IL, United States of America; 7 Department of Animal Sciences, University of Illinois at Urbana-Champaign, Urbana, IL, United States of America; Institute of Tibetan Plateau Research, China

## Abstract

Microbial communities in the rhizosphere make significant contributions to crop health and nutrient cycling. However, their ability to perform important biogeochemical processes remains uncharacterized. Here, we identified important functional genes that characterize the rhizosphere microbial community to understand metabolic capabilities in the maize rhizosphere using the GeoChip-based functional gene array method. Significant differences in functional gene structure were apparent between rhizosphere and bulk soil microbial communities. Approximately half of the detected gene families were significantly (p<0.05) increased in the rhizosphere. Based on the detected *gyrB* genes, Gammaproteobacteria, Betaproteobacteria, Firmicutes, Bacteroidetes and Cyanobacteria were most enriched in the rhizosphere compared to those in the bulk soil. The rhizosphere niche also supported greater functional diversity in catabolic pathways. The maize rhizosphere had significantly enriched genes involved in carbon fixation and degradation (especially for hemicelluloses, aromatics and lignin), nitrogen fixation, ammonification, denitrification, polyphosphate biosynthesis and degradation, sulfur reduction and oxidation. This research demonstrates that the maize rhizosphere is a hotspot of genes, mostly originating from dominant soil microbial groups such as Proteobacteria, providing functional capacity for the transformation of labile and recalcitrant organic C, N, P and S compounds.

## Introduction

The rhizosphere is a unique ecological niche that shapes microbial community structure through the interactions of plant species, root exudates, soil properties, and many other factors [1], [2]. Organic substances released from roots may support high microbial biomass and metabolic activity and thus the assembly of more active and distinct microbial communities in the rhizosphere as compared to those in the bulk soil. Interactions between plants and microorganisms in the rhizosphere control important biogeochemical processes, such as nutrient cycling and greenhouse gas emissions. While some pathogenic microbes undermine plant health, many beneficial microbes in the rhizosphere provide plants with mineral nutrients, phytohormones, and also help to protect the plant against phytopathogens [1], [3]. Thus, characterization of the functional potential of rhizosphere microbial communities is important in order to link soil community activity to plant growth and health.

Previous studies show that the activities of many enzymes are higher in the rhizosphere than in bulk soil [4], due to higher microbial activity and enzymes secreted from roots and microorganisms. Biochemical measurements of soil enzyme activities provide general information, but little is known about the origin of various types of enzyme. With high throughput pyrosequencing-based approaches [5] and microarray-based metagenomic technologies, e.g., GeoChip [6], [7], the composition, structure, and metabolic potential of microbial communities can be rapidly analyzed. For example, GeoChip 3.0 covers ca. 57,000 gene sequences in >292 gene families, allowing quantification of hundreds and thousands of functional genes from known microbes in each sample [7]. Thus, the phylogenetic nature of the data acquisition enables the assignment of metabolic capabilities to specific microbial groups in each niche. Such studies bridge the gap between microbial diversity and potential enzyme activities, which have both been previously investigated separately in the rhizosphere [1]–[3].

Since maize (*Zea mays*) is an important crop, comprehensive phylogenetic investigations of its rhizosphere microbiome have been conducted using high throughput pyrosequencing techniques [5], [8] and other methods [9], [10]. Previous research shows that the rhizosphere of maize is preferentially colonized by Proteobacteria, Bacteroidetes, and Actinobacteria [5], and these organisms may be active in utilizing plant exudates and organic polymers. However, it is important to determine which microorganisms are active in the rhizosphere and what functional genes and pathways are present.

The objective of this study, was to identify important functional genes characterizing the rhizosphere microbial community using a GeoChip-based functional gene array to understand metabolic capabilities in the maize rhizosphere. We hypothesize that the rhizosphere of maize is a hotspot of genes, mostly originating from dominant microbial groups, and providing functional capacity for the transformation of labile and recalcitrant organic C, N, P and S compounds.

## Materials and Methods

### Field description and sampling

Maize (*Zea mays*) was grown near the University of Illinois (Champaign, IL, USA, 40°03′N, 88°12W, 230 m elevation) in a soil classified as a fine-silty, mesic Typic Endoaquoll formed from loess and silt parent material. The field site is a typical arable soil in the U.S. Corn Belt, and it has been in continuous cultivation for over 100 years. The *Z. mays* cultivar cv 34B43 (Pioneer Hi-Bred International, Des Moines, IA, USA) was planted in May following standard agricultural practices in this region with annual applications of 20 g m^−2^ of mixed urea, ammonia, and nitrate fertilizer at planting. No specific permissions were required for research activities in this location, and the field studies did not involve endangered or protected species.

Samples were collected in August 4, 2009, which represents the early reproductive (R) growth stages (the R1–R2 or blister growth stage defined by Hanway [11]). Our rationale for sampling at this time is that it allows us to assess the cumulative growing season influence of the plant on its rhizosphere community at an important early stage in the development of the cereal corn kernels. Thus, this growth period should provide the clearest link between rhizosphere community composition/activity and yield-relevant plant health. However, it should be remembered that the microbial communities are dynamic over the growing season, and this sampling only provides a snapshot view of rhizosphere communities at this important growth stage.

To collect rhizosphere samples, plants were carefully excavated using a shovel, and the soil loosely attached to the root was removed. Fresh roots were collected into a 50 ml centrifuge tube, rinsed in phosphate buffer solution, shaken by hand and centrifuged at 8000 rpm for 10 min. Bulk soil cores of 0–30 cm depth were also collected between sampled plants for paired comparison to the rhizosphere samples. Triplicate samples were taken for both rhizosphere and bulk soil, in which each individual sample was a pool of four plants or soil cores, and each plant or soil core was on average 1 m apart. All the samples were transferred to the lab on dry ice, pre-processed and stored at −80°C for downstream applications. Soil total organic carbon and nitrogen were measured using an element analyzer.

### GeoChip analysis

Soil DNA was extracted by freeze-grinding mechanical lysis as described previously [12]. To determine the abundance of functional genes in the rhizosphere and bulk soils, GeoChip 3.0 was used. This gene array covers ca. 57,000 gene sequences in >292 gene families [7]. For GeoChip analysis, genomic DNA was first amplified using the Templiphi kit (GE Healthcare, NJ) with 100 ng community DNA as the template. Three micrograms of amplified DNA were used for each hybridization. Three technical replicates of each rhizosphere and bulk soil sample were used for microarray hybridization, for a total of 18 hybridizations. Details for template amplification, labeling and hybridization, image processing is described in detail by He et al. [7].

Microarray data were analyzed using the data analysis pipeline developed at the University of Oklahoma (http://ieg.ou.edu/microarray/). Briefly, signal intensity of good quality microarray spots was extracted. Normalized intensity of each gene was calculated by dividing the signal intensity by the mean intensity of the microarray. The matrices of microarray data resulting from our pipeline were considered as ‘species’ abundance in statistical analyses. Microbial diversity indices were analyzed with the Diversity Index Calculator (http://www.reading.ac.uk/ssc/software/diversity/diversity.html). Differences in signal intensity between bulk soil and rhizosphere were tested with two-tailed paired t-tests. All of the original microarray signal data were deposited at NCBI Gene Expression Omnibus (GEO) under accession number GSE61338.

## Results

### Overall differences of microbial communities between rhizosphere and bulk soil

A total of 6201 genes were detected by GeoChip in all samples, with 5777 genes (93.2% of total 6201 genes) enumerated in the rhizosphere and 1983 genes (32.0%) enumerated in the bulk soils. In the rhizosphere, 5390 genes were from bacteria, 103 from archaea and 246 from fungi. In the bulk soil, 1849 genes were from bacteria, 38 from archaea and 84 from fungi. The detected genes were distributed across 248 gene families, of which 53 gene families were only detected in the rhizosphere. Among them, 38 gene families were related to organic compound utilization and degradation. In addition, two gene families were only detected in the bulk soil, and both were related to organic transformation. Gene abundances of 140 gene families were significantly (p<0.05) increased in the rhizosphere (Table S1).

**Figure 1:**
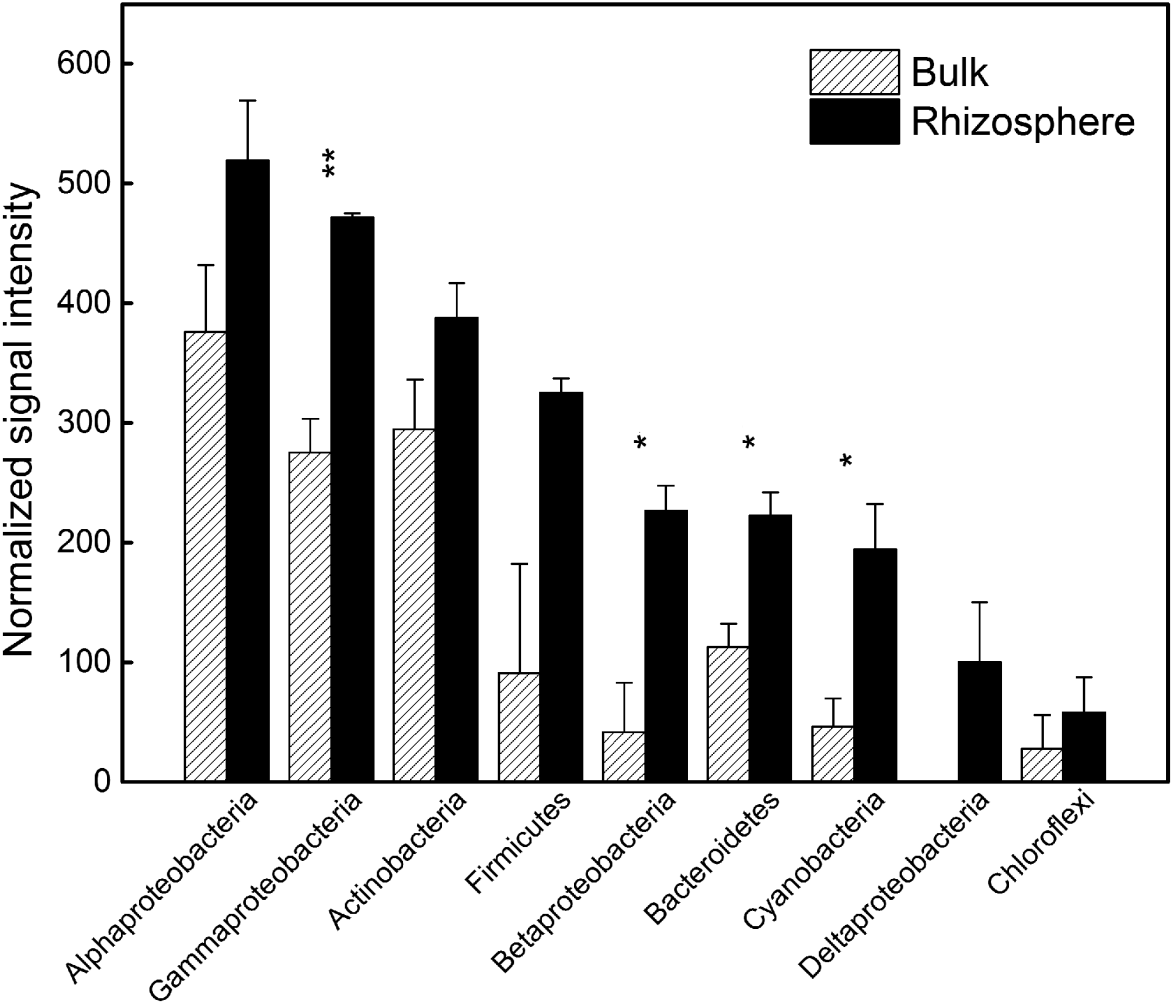
Normalized signal intensity of *gyrB* genes derived from different phylogenetic groups in the rhizosphere and bulk soil. All data are presented as means ± standard errors (n = 3). *p*<*0.05, and **p*<*0.01.

GeoChip 3.0 includes the DNA gyrase subunit B gene (*gyrB*) [7] as a phylogenetic marker to characterize the diversity, composition and structure of microbial communities. A total of 164 *gyrB* probes showed positive signals in both bulk soil and rhizosphere samples. These *gyrB* probes detected were primarily related to Gammaproteobacteria, Betaproteobacteria, Firmicutes, Bacteroidetes and Cyanobacterial phyla (Fig. 1). Signal intensities were higher in the rhizosphere than in the bulk soil. Also, the diversity of functional genes calculated from GeoChip data was significantly higher in the rhizosphere (p<0.05) (Table 1). This is in contrast to the microbial species diversity based on pyrosequencing of the 16S rRNA gene, which was lower in the rhizosphere than that in the bulk soil [5].

**Table 1:** Soil carbon and nitrogen content (%) and microbial diversity indices based on 454-pyrosequencing and GeoChip data in the rhizosphere and bulk soils.

	Bulk soil	Rhizosphere	Significant test
Total carbon	2.26±0.11	2.43±0.08	*
Total nitrogen	0.18±0.01	0.19±0.01	
454 Pyrosequencing data			
Shannon’s diversity index	6.79±0.05	6.29±0.11	**
Shannon’s evenness	0.94±0.01	0.90±0.01	**
GeoChip data			
Shannon’s diversity index	6.56±0.45	7.86±0.30	*
Shannon’s evenness	0.95±0.01	0.96±0.004	

a) Pyrosequencing data are from Li et al. [5]. Samples were collected from the same field site in different years.

b) The value is the average of triplicate samples ± standard deviation.

*p<0.05,

**p<0.01.

Even though the GeoChip array may have some bias in probe selection, the above results showed the maize rhizosphere microbial community had distinct structure and metabolic potential compared to those from the bulk soil. Though many different types of genes were detected, we were especially interested in microbial genes important to nutrient cycling and microbial interactions, such as antibiotic genes and their phylogenetic origins, which were further examined below.

### Autotrophic CO_2_ fixation

**Figure 2:**
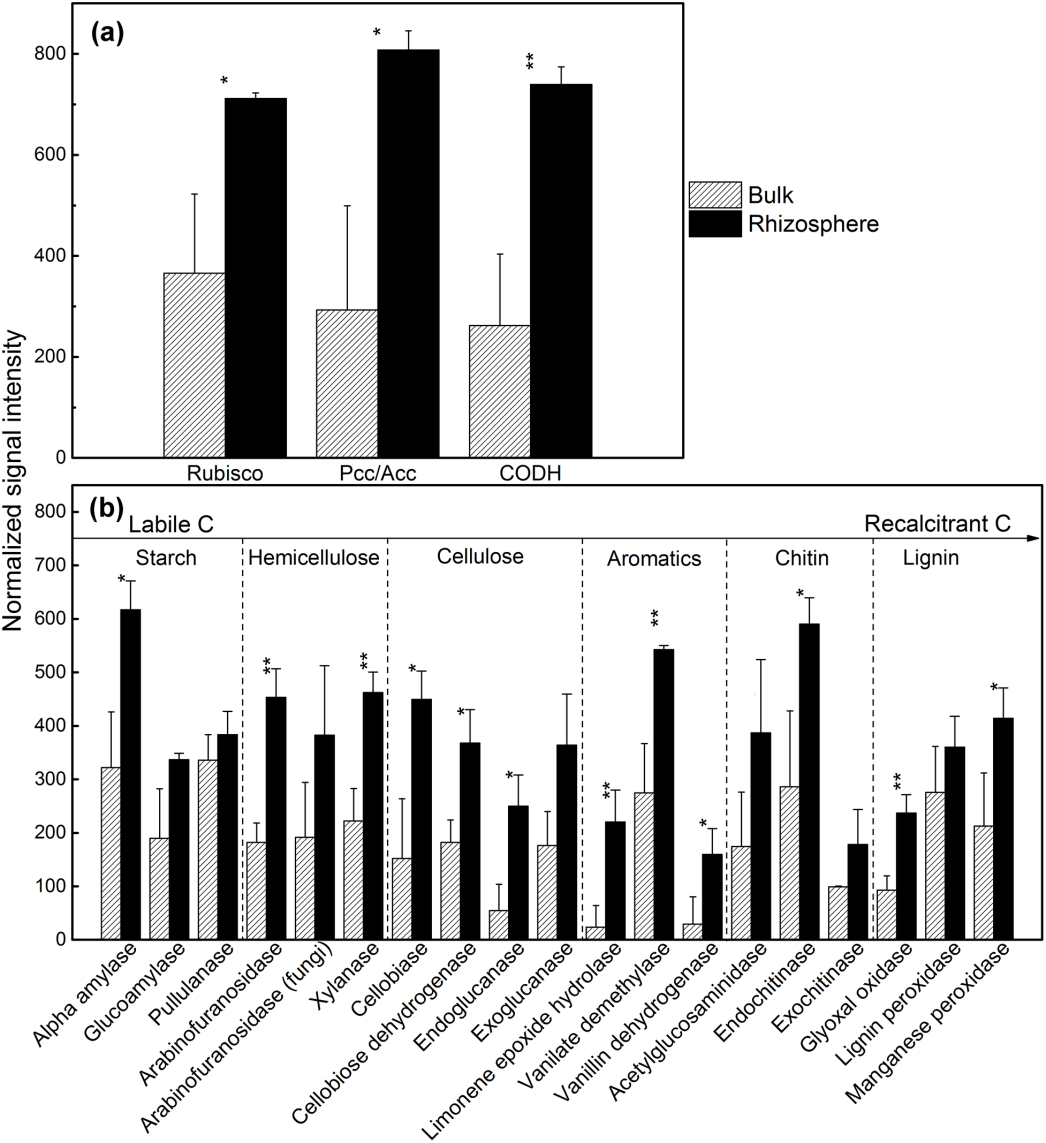
Normalized signal intensity of genes involved in (a) carbon fixation, (b) carbon degradation pathways in the rhizosphere and bulk soil. All data are presented as means ± standard errors (n = 3). *p*<*0.05, and **p*<*0.01.

GeoChip 3.0 contains probes for detecting genes encoding key enzymes involved in autotrophic carbon fixation pathways: ribulose-l,5-bisphosphate carboxylase/oxygenase (Rubisco) for Calvin cycle, propionyl-CoA/acetyl-CoA carboxylase (Pcc/Acc) for 3-hydroxypropionate/malyl-CoA cycle, carbon monoxide dehydrogenase (CODH) for reductive acetyl-CoA pathway, and ATP citrate lyase (AclB) for reductive acetyl-CoA pathway [7]. The Pcc/Acc, Rubisco and CODH pathways were identified to be dominant in both the bulk soil and rhizosphere samples (Fig. 2), and their relative intensity was higher (p<0.05) in the rhizosphere than that in bulk soil, suggesting higher capacity in CO_2_ fixation in the rhizosphere. The AclB pathway was not detected in any of the samples. The CODH genes were mainly distributed in Alphaproteobacteria phylum in both rhizosphere and bulk soil, especially the genera *Rhodopseudomonas* and *Stappia* (Table S2). Also, Rubisco genes were mainly found in Proteobacteria in both rhizosphere and bulk soil, especially the genera *Burkholderia* and *Magnetospirillum*. The *pcc*/*acc* genes for autotrophic carbon fixation were identified mainly in Proteobacteria and Actinobacteria in both rhizosphere and bulk soil, especially the genera *Verminephrobacter*, and *Mycobacterium*, respectively. In contrast, the *pcc*/*acc* genes related to genera *Bradyrhizobium* and *Caulobacter* were only detected in the rhizosphere (Table S3).

### Carbon degradation

Rhizosphere soil had more organic C than bulk soil (Table 1). Among 248 detected gene families, 176 gene families were involved in C degradation and organic matter remediation. These genes included those for the degradation of labile C, e.g., starch (alpha-amylase), hemicelluloses (arabinofuranosidase and xylanase), cellulose (cellobiase, cellobiose dehydrogenase and endoglucanase, mainly in Actinobacteria, Alphaproteobacteria and fungi), aromatics (limonene epoxide hydrolase, vanillate monooxygenase and vanillin dehydrogenase, mainly in Proteobacteria, e.g. *Sphingomonas*, *Bradyrhizobium* and *Pseudomonas*), chitin (endochitinase, mainly in fungi and Bacteroidetes), and lignin (manganese peroxidase and glyoxal oxidase, mainly in fungi, especially the class Basidiomycetes) (Fig. 2).

### Nitrogen cycling

**Figure 3:**
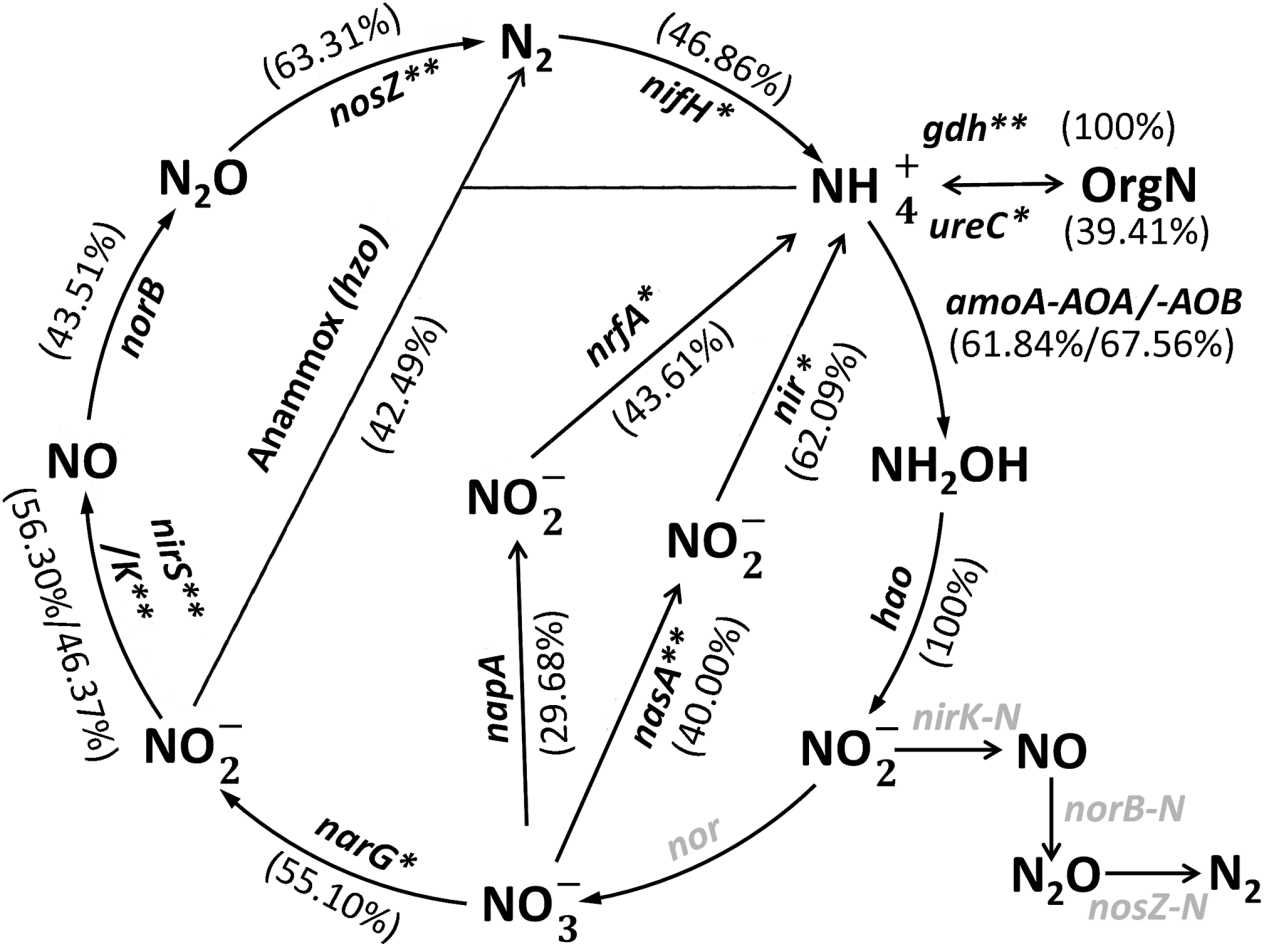
Differences in the abundance of N cycling genes in the rhizosphere and bulk soil. The numbers in brackets indicate the percentage difference of a functional gene signal intensity between rhizosphere and bulk soil samples relative to the normalized signal intensity in the bulk soil sample. The gray-colored genes were not detected by GeoChip 3.0. *p*<*0.05, and **p*<*0.01.

Total soil N levels were similar in the maize rhizosphere and in the bulk soil (Table 1). GeoChip detected genes critical to all the major pathways in the N cycle. Most of genes were more abundant in the rhizosphere than bulk soil (Fig. 3).

Firstly, *nifH* for N fixation was significantly (p<0.05) enriched in the rhizosphere compared to the bulk soil (Table S2 and S3). The most abundant *nifH* genes detected in all samples were from uncultured microorganisms (80–87% of total *nifH* hybridization signal intensities), which suggests that many N_2_-fixing microorganisms are still unknown. Among four defined clusters of *nifH* genes, cluster I genes were most represented (6% of total *nifH* signal in rhizosphere and 12% in bulk soil), especially those from *Rhodobacter* and *Rhodopseudomonas*.

Secondly, two genes encoding key enzymes (glutamate dehydrogenase/*gdh* and urease/*ureC*) for ammonification (N mineralization) were significantly (p<0.05) enriched in the maize rhizosphere compared to bulk soil (Table S2 and S3). *Gdh* genes were only detected in the rhizosphere, mainly distributed in the bacterial phylum Actinobacteria and archaeal genus *Thermoplasma*, while *ureC* genes were mainly distributed in the bacterial phyla Proteobacteria and Actinobacteria.

Thirdly, two genes encoding key enzymes (ammonia monooxygenase/*amoA* and hydroxylamine oxidoreductase/*hao*) for nitrification were detected. Both of the bacterial and archaeal *amoA* genes were dominant in the rhizosphere. The *hao* genes were only detected in the rhizosphere and largely derived from Rhodobacteraceae.

Fourthly, two key genes (nitrate reductase/*napA* and c-type cytochrome nitrite reductase/*nrfA*) for dissimilatory N reduction to ammonium (DNRA) were significantly (p<0.05) overrepresented in the rhizosphere compared to bulk soil, mainly derived from *Desulfitobacterium* and *Syntrophobacter*. Also, three genes (*nasA, nirA* and *nirB*) for assimilatory N reduction to ammonium were significantly (p<0.05) enriched in the rhizosphere compared to bulk soil. The *nasA* gene was mainly found in the uncultured bacteria, the *nirA* gene in uncultured archaea, and the *nirB* gene in Verrucomicrobiae, Actinomycetales, and genus *Pseudomonas*.

In addition, the abundances of five key genes (*narG*, *nirS*/*nirK*, *norB* and *nosZ*) involved in the denitrification process were significantly higher (p<0.05) in the rhizosphere than in the bulk soil. Most of these genes were found in uncultured bacteria. Some of *narG* genes detected in the maize rhizosphere were indicated in Actinobacteria and Proteobacteria, which is consistent with a previous report [13].

### Phosphorus utilization

**Figure 4:**
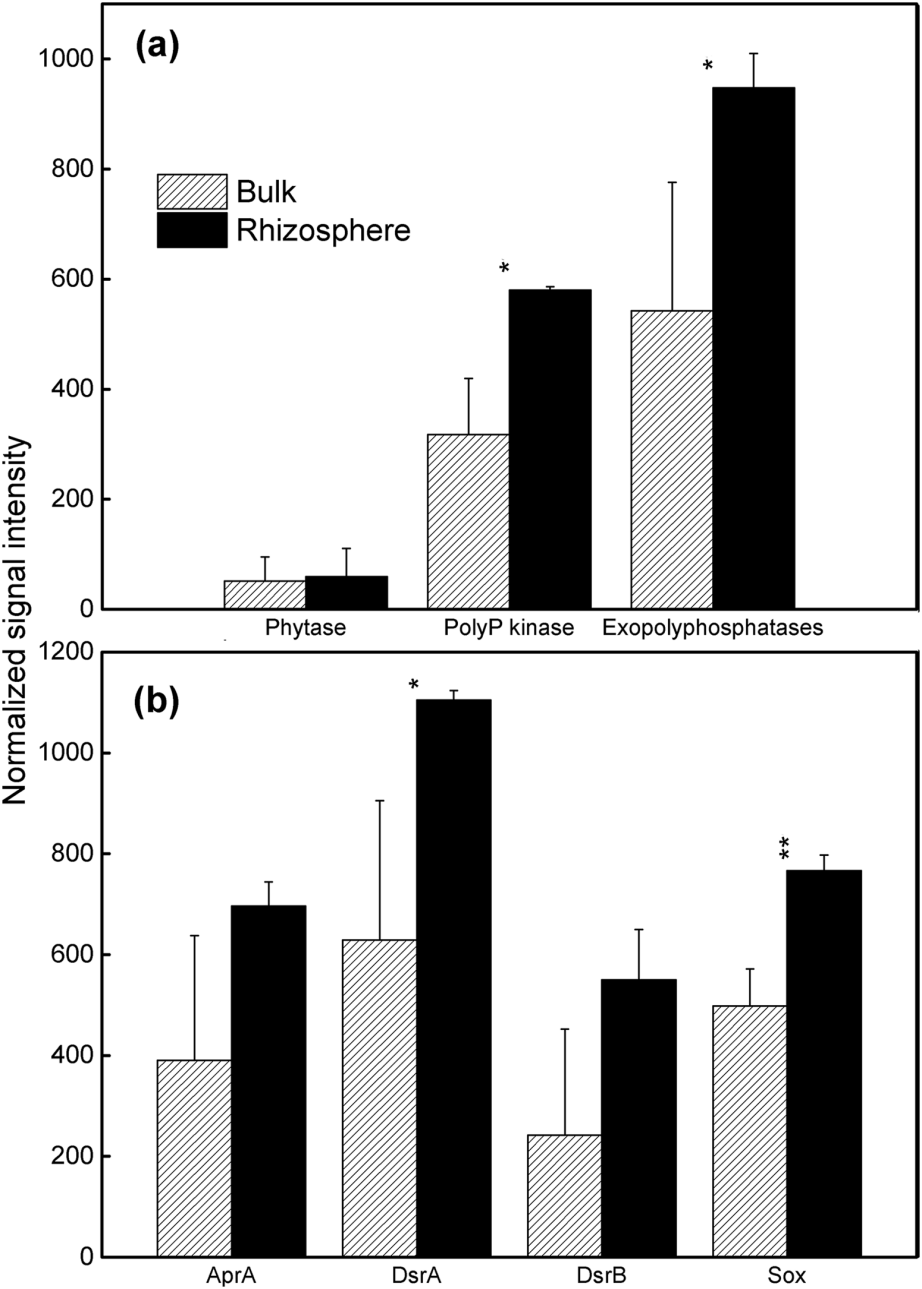
Normalized signal intensity of genes involved in (a) phosphorus utilization, (b) sulfur cycling in the rhizosphere and bulk soil. All data are presented as means ± standard errors (n = 3). *p*<*0.05, and **p*<*0.01.

GeoChip 3.0 contains probes targeting phosphorus utilization, exopolyphosphatase (Ppx) for inorganic polyphosphate degradation, polyphosphate kinase (Ppk) for polyphosphate biosynthesis in prokaryotes, and phytase for phytate degradation [7]. The total signal intensity of *ppx* and *ppk* genes was significantly (p<0.05) increased in the rhizosphere (Fig. 4), suggesting a possible enhancement of polyphosphate transformation and the availability of inorganic P in the maize rhizosphere. These genes were mainly distributed in Proteobacteria and Actinobacteria. The abundance of phytase genes, mainly derived from fungi *Ceriporia* sp. in the rhizosphere and *Obesumbacterium proteus* in bulk soil, was not significantly different between rhizosphere and bulk soil, indicating that organic P degradation capability may be not significantly different.

### Sulfur cycling

Four enzymes involved in the sulfur cycling of microbial communities are included in GeoChip 3.0: sulfite reductase (DsrA/DsrB) for sulfur reduction, sulfite oxidase (Sox) for sulfur oxidation, dissimilatory adenosine-5′-phosphosulfate reductase (AprA) for both microbial sulfate reduction and sulfur oxidation processes [7]. The total signal intensity of *dsrA* and *sox* genes was significantly (p<0.05) increased in the rhizosphere (Fig. 4), suggesting a possible increase in the sulfur transformation rate. *sox* genes were mainly derived from Alphaproteobacteria, especially the order Rhizobiales, while the other three genes were mainly affiliated with Deltaproteobacteria.

### Antibiotic resistance

**Figure 5:**
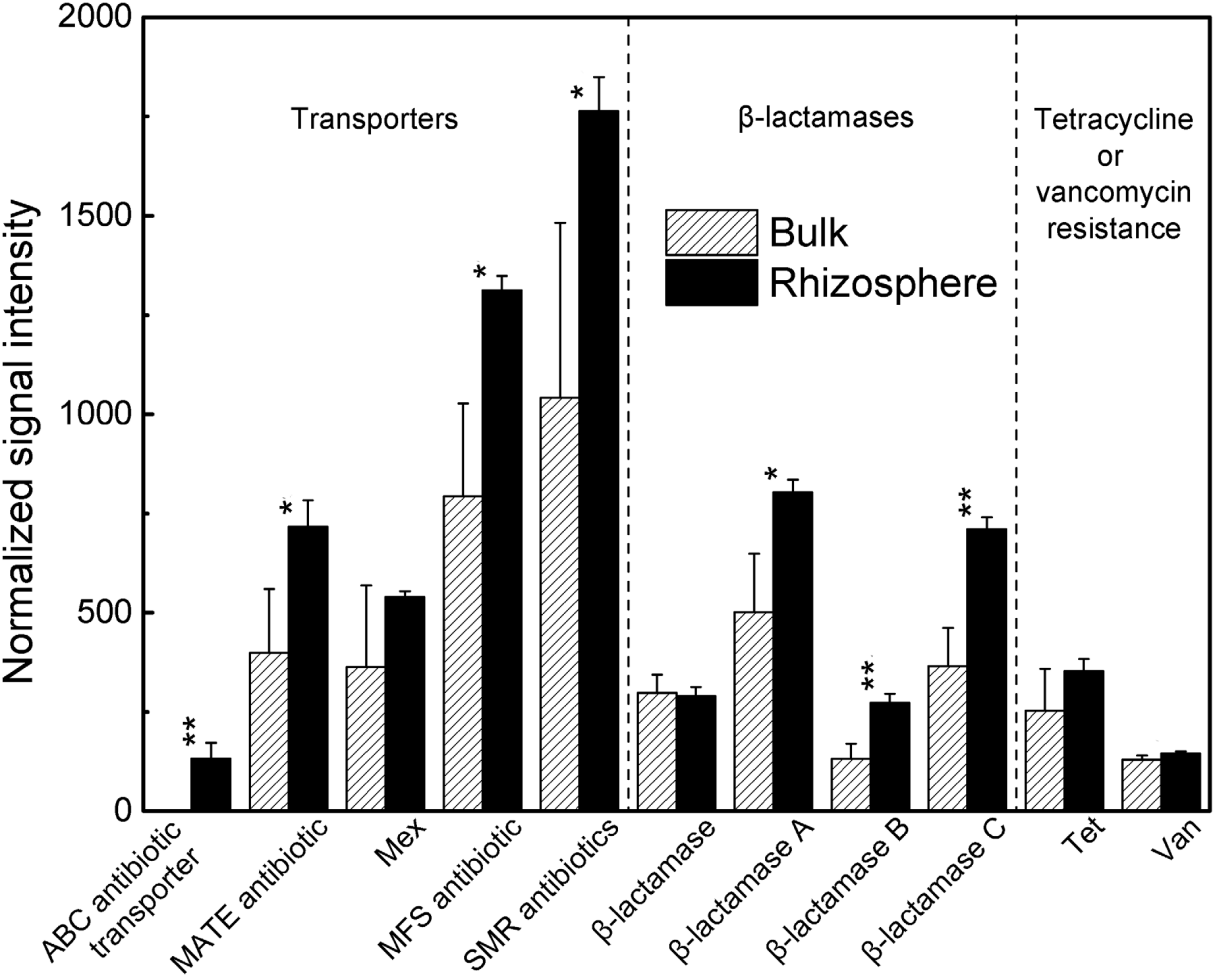
Normalized signal intensity of antibiotic resistance genes in the rhizosphere and bulk soil. All data are presented as means ± standard errors (n = 3). *p*<*0.05, and **p*<*0.01.

Eleven genes for antibiotic resistance are included in GeoChip 3.0: five for transporters, four for β-lactamase genes, and two for tetracycline and vancomycin resistance [7]. We found most of these genes to be significantly more abundant in the rhizosphere than in bulk soil (Fig. 5; p<0.05). The small multidrug resistance gene family (SMR) showed the highest signal intensity among these five transporter genes, which were mainly affiliated with genera *Mycobacterium*, *Bacillus* and *Yersinia* in both rhizosphere and bulk soil. Major facilitator super gene family (MFS) also showed high signal intensity after SMR, and they were mainly derived from the genus *Burkholderia*. The ABC antibiotic transporter was only detected in the rhizosphere.

## Discussion

GeoChip-based analysis in this study reveals the assemblage of dominant microbiomes from an explicitly functional perspective rather than from taxonomic classification only. Our previous study showed that the maize rhizosphere had significantly reduced bacterial species diversity in comparison to bulk soil [5]. In contrast, GeoChip analysis revealed that functional diversity increased in the rhizosphere (Table 1). Root exudates are driving forces to determine the assemblage of rhizosphere microbial community [14], [15]. There are a wide variety of compounds, such as organic acids, sugars, amino acids, lipids, enzymes and aromatics, in the root exudates [1]. Half of the detected gene families were significantly more abundant in the rhizosphere than those in the bulk soil, suggesting that a broad diversity of metabolic activities is enhanced in the rhizosphere. Rhizosecretion (exudate) may activate the growth of previously dormant microbes and their metabolic potentials, including fast-growing r-strategist microbes [14]. The r-strategists may respond rapidly to the freshly input organic matter by increasing their growth, functional gene expression and decomposition rates [16].

Enzyme activities involved in carbon and nitrogen cycling are influenced by plant-microorganism-fauna interactions [17]. Geochip data in this study showed that the changes in the structure and metabolic potential of rhizosphere microbial communities were reflected in a significant increase in the abundance of many functional genes compared to the bulk soil. The higher abundances of functional genes (e.g., CODH, xylanase, glyoxal oxidase and vanilate demethylase) in the rhizosphere suggests that plants select functional groups with a strong capacity for C source utilization. We speculate that the rhizosphere may favor those decomposers for labile C compounds and plant polymers since the gene abundances responsible for the degradation of these organic substrates were significantly over-represented in the rhizosphere. The ability to carry out both simple and complex carbohydrate catabolism was mainly detected in Proteobacteria, Actinobacteria, Firmicutes, and fungi (Table S3), whereas the ability to catabolize complex aromatic compounds was indicated mainly by Proteobacteria and fungi. The increased potential to transform lignin-like compounds and other naturally occurring polyaromatics in the rhizosphere might be related to manganese peroxidase found in Basidiomycetes [18].

A previous study showed that increased microbial activity and nitrogen mineralization were coupled to the changes in microbial community structure in the rhizosphere of Bt corn [19]. The stimulatory effect of maize roots on denitrification was observed at both high and low soil nitrate levels [20]. Our GeoChip results suggested that N fixation, ammonification, denitrification, and N reduction were significantly higher in the maize rhizosphere compared to the bulk soil, especially by members of Proteobacteria (Table S3), possibly leading to more active microbially mediated N transformation in the rhizosphere. GeoChip analysis further showed that the genes involved in C, N, P, S cycling stimulated by the maize rhizosphere were mainly found in organisms belonging to the phyla Proteobacteria and Actinobacteria. This is also supported by the finding that the maize rhizosphere was preferentially colonized by Proteobacteria and Actinobacteria [5]. Within these phyla, the genera *Bradyrhizobium*, *Pseudomonas*, *Rhodopseudomonas* and *Mycobacterium* were overrepresented in the rhizosphere, and these genera harbored diverse functional genes for nutrient transformation. GeoChip data showed that *Bradyrhizobium* played important roles in autotrophic carbon fixation and aromatic degradation; *Pseudomonas* involved in aromatics degradation and assimilatory N reduction to ammonium; *Rhodopseudomonas* involved in autotrophic carbon fixation and N fixation; *Mycobacterium* involved in autotrophic carbon fixation and antibiotic resistance. A metaproteomics study also revealed that bacterial proteins were mostly linked to the Proteobacteria and Actinobacteria in the rhizosphere of rice [21], suggesting that these two bacterial phyla constitute important parts of ‘core rhizosphere microbiome’ that maintains various normal functions and are also effective in controlling soil-borne pathogens.

Since the rhizosphere processes are driven by complex interactions between roots, micro-organisms and soil fauna [17], higher competition for mineral elements in the rhizosphere than bulk soil is expected. GeoChip data supported the hypothesis that microbial competition in the rhizosphere might be greater than that found in bulk soil, as indicated by the higher abundance of antibiotic resistance genes in the rhizosphere. Some microorganisms produce unique antimicrobial metabolites toxic to other microorganisms, allowing them to colonize and proliferate on plant surfaces in the presence of other microbial communities. The genera *Burkholderia, Mycobacterium*, *Bacillus* and *Yersinia* were found to be important antibiotic producers in this study, corroborating their abundant distributions in the rhizosphere of many crops, such as maize [1], [5]. The higher occurrence of antibiotic resistance genes also provide the frontline defense for plant roots against attack by soilborne pathogens [22]. Microbes that can degrade or detoxify these metabolites via specific functional genes gain a competitive advantage [1].

With GeoChip-based functional gene arrays, we have been able to identify functional potentials with the most relevant focus on functional ecology in the rhizosphere of maize. The data supports the concept that the rhizosphere of maize is a hotspot of genes for the transformation of labile and recalcitrant organic C, N, P and S compounds. These functional genes mostly originate from dominant microbial groups, such as Proteobacteria. This study provides comprehensive baseline information on functional capacity in the maize rhizosphere. Specific plant-microbe interactions should be further defined by linking the plant C metabolism by microbial species with a particular functional role in the rhizosphere [2].

## Supporting Information

Table S1 **Normalized signal intensity of gene families significantly different in the rhizosphere and bulk soil (p<0.05).**
(XLSX)

Table S2 **The dominant genes affiliated to different genera.**
(XLSX)

Table S3 **Log_10_ values of normalized signal intensity of dominant gene categories affiliated to each phylum.**
(XLSX)
